# Non contiguous-finished genome sequence and description of *Bacillus jeddahensis* sp. nov.

**DOI:** 10.1186/s40793-015-0024-y

**Published:** 2015-08-01

**Authors:** Fadi Bittar, Fehmida Bibi, Dhamodharan Ramasamy, Jean-Christophe Lagier, Esam I. Azhar, Asif A. Jiman-Fatani, Ahmed K. Al-Ghamdi, Ti Thien Nguyen, Muhammad Yasir, Pierre-Edouard Fournier, Didier Raoult

**Affiliations:** URMITE, Aix-Marseille Université, Faculté de médecine, Marseille, France; Special Infectious Agents Unit, King Fahd Medical Research Center, King Abdulaziz University, Jeddah, Saudi Arabia; Department of Medical Laboratory Technology, Faculty of Applied Medical Sciences, King Abdulaziz University, Jeddah, Saudi Arabia; Department of Medical Microbiology and Parasitology, Faculty of Medicine, King Abdulaziz University, Jeddah, Saudi Arabia

**Keywords:** *Bacillus jeddahensis*, Genome, Taxono-genomics, Culturomics, Human feces

## Abstract

Strain JCE^T^ was isolated from the fecal sample of a 24-year-old obese man living in Jeddah, Saudi Arabia. It is an aerobic, Gram-positive, rod-shaped bacterium. This strain exhibits a 16S rRNA nucleotide sequence similarity of 97.5 % with *Bacillus niacini*, the phylogenetically closest species with standing nomenclature. Moreover, the strain JCE^T^ presents many phenotypic differences, when it is compared to other *Bacillus* species, and shows a low MALDI-TOF Mass Spectrometry score that does not allow any identification. Thus, it is likely that this strain represents a new species. Here we describe the features of this organism, together with the complete genome sequence and annotation. The 4,762,944 bp long genome (1 chromosome but no plasmid) contains 4,654 protein-coding and 98 RNAs genes, including 92 tRNA genes. The strain JCE^T^ differs from most of the other closely *Bacillus* species by more than 1 % in G + C content. In addition, digital DNA-DNA hybridization values for the genome of the strain JCE^T^ against the closest *Bacillus* genomes range between 19.5 to 28.1, that confirming again its new species status. On the basis of these polyphasic data made of phenotypic and genomic analyses, we propose the creation of *B*acillus *jeddahensis* sp. nov*.* that contains the strain JCE^T^.

**Electronic supplementary material:**

The online version of this article (doi:10.1186/s40793-015-0024-y) contains supplementary material, which is available to authorized users.

## Introduction

Currently, a polyphasic approach that combines proteomic by MALDI-TOF spectra analysis, genomic data and phenotypic characterization is used widely to describe new bacterial species [1–13].

The genus Bacillus, described by Cohn [14] more than 140 years ago, includes actually 310 species names (296 validly and 14 not-validly published species) [15]. Species belonging to this genus are Gram-positive or variable and mostly motile and spore-forming bacteria. Bacillus spp. are ubiquitous bacteria isolated from various environmental sources but it could be involved in human infections [16].

Strain JCE^T^ (= CSUR P732 = DSM 28281) is the type strain of Bacillus*jeddahensis* sp. nov. This bacterium is a Gram-positive, flagellated, facultatively anaerobic, indole-negative bacillus that has rounded-ends. It was isolated from the stool sample of a 24-year-old obese man living in Jeddah, Saudi Arabia as part of a culturomics study aiming at cultivating bacterial species within human feces. By applying large scale of culture conditions, culturomics allowed previously the isolation of many new bacterial species from human stool samples [17–19].

Here we present a summary classification and a set of features for *B. jeddahensis* sp. nov. strain JCE^T^ together with the description of the complete genome sequence and annotation. These characteristics support the circumscription of the species *B. jeddahensis* [20].

## Organism information

### Classification and features

**Fig. 1:**
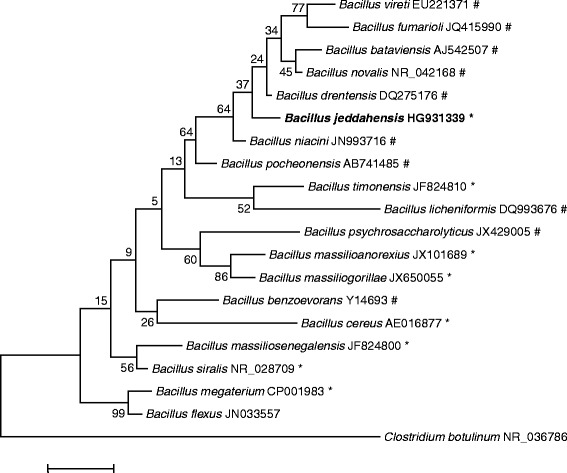
Phylogenetic tree highlighting the position of Bacillus *jeddahensis* strain JCE^T^ relative to other type strains within the Bacillus genus. GenBank accession numbers are indicated in parentheses. Sequences were aligned using MUSCLE, and phylogenetic inferences obtained using the maximum-likelihood method and Kimura 2-parameter model within the MEGA 6 software [50]. Numbers at the nodes are percentages of bootstrap values obtained by repeating the analysis 1,000 times to generate a majority consensus tree. *Clostridium botulinum* was used as outgroup. The scale bar represents a rate of substitution per site of 0.01. *indicates the strains used in the tree have a sequenced genome. # indicates that a sequenced genome is available for this species but not for the strain used to build the tree

In April 2013, a fecal sample was collected from a 24-year-old obese (body mass index 52 kg/m2) man living in Jeddah, Saudi Arabia (Table 1). Written assent was obtained from this individual. Both the study and the assent procedure were approved by Ethical Committee of the King Abdulaziz University, King Fahd medical Research Centre, Saudi Arabia (agreement number 014-CEGMR-2-ETH-P) and the Ethical Committee of the Institut Fédératif de Recherche IFR48, Faculty of Medecine, Marseille, France (agreement numbers 09–022 and 11–017). The fecal specimen was preserved at −80 °C after collection and sent to Marseille. Strain JCE^T^ (Table 1) was isolated in July 2013 by cultivation on blood culture bottle (Becton Dickinson, Temse, Belgique) supplemented with rumen fluid and sheep blood. This strain exhibited a 97.5 % 16S rRNA nucleotide sequence similarity with Bacillus niacini, the phylogenetically closest validly published Bacillus species (Fig. 1), when it was compared against NCBI database and Ribosomal Database Project. This value was equal to the percentage of 16S rRNA gene sequence threshold recommended by Meier-Kolthoff et al. for Firmicutes to delineate a new species without carrying out DNA-DNA hybridization with maximum error probability of 0.01 % [21].

**Table 1:** Classification and general features of *B*acillus* jeddahensis* strain JCE^T^

MIGS ID	Property	Term	Evidence code^a^
	Current classification	Domain: *Bacteria*	TAS [39]
		Phylum: *Firmicutes*	TAS [40–42]
		Class: *Bacilli*	TAS [43, 44]
		Order: *Bacillales*	TAS [45, 46]
		Family: *Bacillaceae*	TAS [45, 47]
		Genus: *Bacillus*	TAS [4, 45, 48]
		Species: *Bacillus jeddahensis*	IDA
		Type strain: JCE^T^	IDA
	Gram stain	Positive	IDA
	Cell shape	Rod-shaped	IDA
	Motility	Non-motile	IDA
	Sporulation	Sporulating	IDA
	Temperature range	Mesophile	IDA
	Optimum temperature	37 °C	IDA
	pH range; Optimum	Not determined	
MIGS-6.3	Salinity	growth in BHI medium + 3 % NaCl	IDA
MIGS-22	Oxygen requirement	Facultative Anaerobic	IDA
	Carbon source	varied (see Additional file 1: Table S1)	IDA
	Energy source	chemoorganoheterotrophic	IDA
MIGS-6	Habitat	Human gut	IDA
MIGS-15	Biotic relationship	Free living	IDA
MIGS-14	Pathogenicity	Unknown	
	Biosafety level	2	NAS
	Isolation	Human faeces	IDA
MIGS-4	Geographic location	Saudi Arabia	IDA
MIGS-5	Sample collection time	July 2013	IDA
MIGS-4.1	Latitude	21° 25' 20.953"	IDA
MIGS-4.1	Longitude	39° 49' 34.262"	IDA
MIGS-4.3	Depth	unknown	
MIGS-4.4	Altitude	unknown	

Evidence codes - *IDA* Inferred from Direct Assay, *TAS* Traceable Author Statement (i.e., a direct report exists in the literature), *NAS* Non-traceable Author Statement (i.e., not directly observed for the living, isolated sample, but based on a generally accepted property for the species, or anecdotal evidence). These evidence codes are from the Gene Ontology project [49]. If the evidence is IDA, then the property was directly observed for a live isolate by one of the authors or an expert mentioned in the acknowledgements

**Fig. 2:**
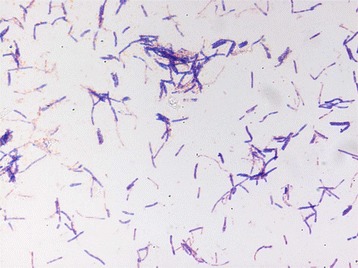
Gram staining of B. jeddahensis strain JCE^T^

**Fig. 3:**
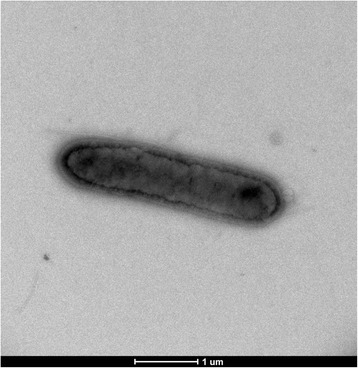
Transmission electron microscopy of B. jeddahensis strain JCET, using a Morgani 268D (Philips) at an operating voltage of 60 kV. The scale bar represents 1 μm

Different growth temperatures (28, 30, 37, 45, 56 °C) were tested. Growth occurred for the temperatures (28–45 °C), but the optimal growth was observed at 37 °C. Colonies were 0.4–0.5 mm in diameter on Columbia agar, appear smooth and grey in color at 37 °C. Growth of the strain was tested under anaerobic and microaerophilic conditions using GENbag anaer and GENbag microaer systems, respectively (BioMérieux), and in aerobic conditions, with or without 5 % CO_2_. Growth was achieved under aerobic (with and without CO_2_), microaerophilic and anaerobic conditions. Gram staining showed Gram positive bacilli (Fig. 2). A motility test was negative. Cells grown on agar sporulate and the rods have a length ranging from 3.83 to 4.71 μm (mean 4.14 μm) and a diameter ranging from 0.75 to 0.95 μm (mean 0.87 μm). Both the length and the diameter were determined by negative staining transmission electron microscopy (Fig. 3).

Strain JCE^T^ exhibited oxidase activity but not catalase activity. Using API 50CH system (BioMerieux), a positive reaction was observed for D-arabinose, L-arabinose, D-xylose, D-glucose, D-fructose, D-mannose, N-acetylglucosamine, esculin, D-maltose, D-trehalose, and weak reaction for D-melezitose. Negative reactions were observed for the remaining carbohydrate tests (i.e. glycerol, erythritol, D-ribose, L-xylose, D-adonitol, methyl-β-D-xylopyranoside, D-galactose, L-sorbose, L-rhamnose, dulcitol, inositol, D-mannitol, D-sorbitol, methyl-α-D-mannopyranoside, methyl-α-D-glucopyranoside, amygdalin, arbutin, salicin, D-cellobiose, D-lactose, D-melibiose, D-saccharose, inulin, D-raffinose, amidon, glycogen, xylitol, gentiobiose, D-turanose, D-lyxose, D-tagatose, D-fucose, L-fucose, D-arabitol, L-arabitol, potassium gluconate, potassium 2-ketogluconate and potassium 5-ketogluconate). Using API ZYM, positive reactions were observed for esterase (C 4), esterase lipase (C 8), acid phosphatase, naphthol-AS-BI-phosphohydrolase and β-glucosidase. Negative reactions were observed for alkaline phosphatase, lipase (C 14), leucine arylamidase, valine arylamidase, cystine arylamidase, trypsin, α-chymotrypsin, α-galactosidase, β-galactosidase, β-glucuronidase, α-glucosidase, N-acetyl-β-glucosaminidase, α-mannosidase and α-fucosidase. Using API NE system, nitrates were reduced to nitrites, the urease reaction, indole production, arginine dihydrolase and gelatin hydrolysis were negative, the following carbon sources were assimilated: D-glucose, D-mannose, N-acetylglucosamine and D-maltose, and the following carbon sources were not assimilated: L-arabinose, D-mannitol, potassium gluconate, capric acid, adipic acid, malic acid, trisodium citrate and phenylacetic acid. *B. jeddahensis* is susceptible to imipenem, doxycyclin amoxicillin, amoxicillin-clavulanate and gentamycin, but resistant to metronidazole, trimethoprim/sulfamethoxazole, rifampicin, vancomycin, erythromycin, ceftriaxone, ciprofloxacin and benzylpenicillin.

When compared to other Bacillus species [18, 22–24], *B. jeddahensis* sp. nov. strain JCE^T^ exhibited the phenotypic differences detailed in Additional file 1: Table S1.

**Fig. 4:**
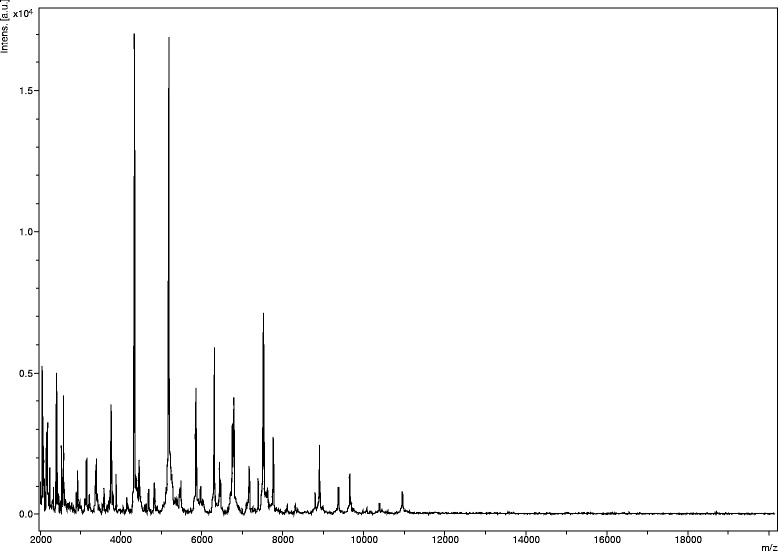
Reference mass spectrum from *B. jeddahensis* strain JCE^T^. Spectra from twelve individual colonies were compared and a reference spectrum was generated

**Fig. 5:**
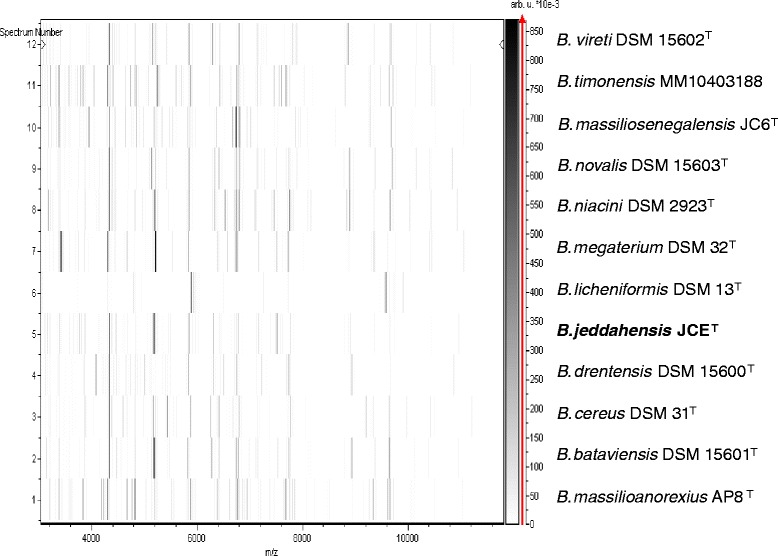
Gel view comparing *Bacillus jeddahensis* JCE^T^ spectra with other members of the *Bacillus* genus (*B. niacini, B. drentensis, B. novalis, B. bataviensis, B. vireti, B. massilioanorexius, B. massiliosenegalensis, B. megaterium, B. timonensis, B. cereus* and *B. licheniformis*). The Gel View displays the raw spectra of all loaded spectrum files arranged in a pseudo-gel like look. The x-axis records the m/z value. The left y-axis displays the running spectrum number originating from subsequent spectra loading. The peak intensity is expressed by a gray-scale scheme code. The color bar and the right y-axis indicate the relation between the color a peak is displayed with and the peak intensity in arbitrary units

Matrix-assisted laser-desorption/ionization time-of-flight (MALDI-TOF) MS protein analysis was carried out as previously described [2] using a Microflex spectrometer (Bruker Daltonics, Leipzig, Germany). Twelve distinct deposits were done for strain JCE^T^ from 12 isolated colonies. The twelve JCE^T^ spectra were imported into the MALDI BioTyper software (version 2.0, Bruker) and analyzed by standard pattern matching (with default parameter settings) against 6,335 bacterial spectra including 210 spectra from 110 Bacillus species, used as reference data, in the BioTyper database. Interpretation of scores was as follows: a score ≥ 2 enabled the identification at the species level, a score ≥ 1.7 but < 2 enabled the identification at the genus level; and a score < 1.7 did not enable any identification (These scores were established by the manufacturer Bruker Daltonics). For strain JCE^T^, the obtained scores ranged from 1.4 to 1.6, thus suggesting that our isolate was not a member of a known species. We incremented our database with the spectrum from strain JCE^T^ (Fig. 4). Spectrum differences with other of Bacillus species are shown in Fig. 5.

## Genome sequencing information

### Genome project history

On the basis of phenotypic characteristics of this strain and because of the low16S rRNA similarity to other members of the genus Bacillus*,* it is likely that the strain represents a new species and thus it was chosen for genome sequencing. It was the 348th genome of a Bacillus species (Genomes Online Database) and the first genome of Bacillus*jeddahensis* sp. nov. sequenced. A summary of the project information is shown in Table 2. The Genbank accession number is CCAS00000000 (Table 2) and consists of 149 contigs. Table 2 shows the project information and its association with MIGS version 2.0 compliance [25].

**Table 2:** Project information

MIGS ID	Property	Term
MIGS-31	Finishing quality	High-quality draft
MIGS-28	Libraries used	Paired end and mate pair
MIGS-29	Sequencing platform	MiSeq Technology (Illumina Inc)
MIGS-31.2	Sequencing coverage	94.91x
MIGS-30	Assemblers	Newbler version 2.5.3
MIGS-32	Gene calling method	Prodigal
	EMBL Date of Release	2014
	EMBL ID	CCAS00000000
MIGS-13	Source material identifier	JCE^T^
	Project relevance	Study of the human gut microbiome

### Growth conditions and genomic DNA preparation

*B. jeddahensis* sp. nov. strain JCE^T^, CSUR P732, DSM 28281, was grown aerobically on 5 % sheep blood-enriched Columbia agar at 37 °C. Four Petri dishes were spread and resuspended in 3 × 500 μl of TE buffer and stored at 80 °C. Then, 500 μl of this suspension were thawed, centrifuged 3 min at 10,000 rpm and resuspended in 3 × 100 μL of G2 buffer (EZ1 DNA Tissue kit, Qiagen). A first mechanical lysis was performed by glass powder on the Fastprep-24 device (Sample Preparation system, MP Biomedicals, USA) using 2 × 20 s cycles. DNA was then treated with 2.5 μg/μL lysozyme (30 min at 37 °C) and extracted using the BioRobot EZ1 Advanced XL (Qiagen). The DNA was then concentrated and purified using the Qiamp kit (Qiagen). The yield and the concentration was measured by the Quant-it Picogreen kit (Invitrogen) on the Genios Tecan fluorometer at 50 ng/μl.

### Genome sequencing and assembly

Genomic DNA of *B. jeddahensis* was sequenced on the MiSeq Technology (Illumina Inc, San Diego, CA, USA) with the 2 applications: paired end and mate pair. The paired end and the mate pair strategies were barcoded in order to be mixed respectively with 14 others genomic projects prepared with the Nextera XT DNA sample prep kit (Illumina) and eleven others projects with the Nextera Mate Pair sample prep kit (Illumina). The DNAg was quantified by a Qubit assay with the high sensitivity kit (Life technologies, Carlsbad, CA, USA) to 16 ng/μl and dilution was performed to require 1ng of each genome as input to prepare the paired end library. The « tagmentation » step fragmented and tagged the DNA. Then limited cycle PCR amplification (twelve cycles) completed the tag adapters and introduced dual-index barcodes. After purification on AMPure XP beads (Beckman Coulter Inc, Fullerton, CA, USA), the libraries were then normalized on specific beads according to the Nextera XT protocol (Illumina). Normalized libraries were pooled into a single library for sequencing on the MiSeq. The pooled single strand library was loaded onto the reagent cartridge and then onto the instrument along with the flow cell. Automated cluster generation and paired end sequencing with dual index reads were performed in a single 39-h run in 2 × 250-bp. Total information of 5.3 Gb was obtained from a 574 K/mm2 cluster density with a cluster passing quality control filters of 95.4 % (11,188,000 clusters). Within this run, the index representation for *B. jeddahensis* was determined to 10.3 %. The 1,062,432 reads were filtered according to the read qualities. The mate pair library was prepared with 1 μg of genomic DNA using the Nextera mate pair Illumina guide. The genomic DNA sample was simultaneously fragmented and tagged with a mate pair junction adapter. The profile of the fragmentation was validated on an Agilent 2100 BioAnalyzer (Agilent Technologies Inc, Santa Clara, CA, USA) with a DNA 7500 labchip. The DNA fragments ranged in size from 1 kb up to 11 kb with an optimal size at 5 kb. No size selection was performed and 600 ng of tagmented fragments were circularized. The circularized DNA was mechanically sheared to small fragments with an optimal at 692 bp on the Covaris device S2 in microtubes (Covaris, Woburn, MA, USA). The library profile was visualized on a High Sensitivity Bioanalyzer LabChip (Agilent Technologies Inc, Santa Clara, CA, USA). The libraries were normalized at 2 nM and pooled. After a denaturation step and dilution at 10 pM, the pool of libraries was loaded onto the reagent cartridge and then onto the instrument along with the flow cell. Automated cluster generation and sequencing run were performed in a single 42-h run in a 2 × 250-bp. Total information of 3.9 Gb was obtained from a 399 K/mm2 cluster density with a cluster passing quality control filters of 97.9 % (7,840,000 clusters). Within this run, the index representation for *B. jeddahensis* was determined to 9.37 %. The 718,848 reads were filtered according to the read qualities. The passed filter sequences were assembled using Newbler with 90 % identity and 40-bp as overlap. The final assembly identified 149 large contigs (>1.5 kb) generating a genome size of 4.76 Mb which corresponds to a genome coverage of 94.91x.

### Genome annotation

Open Reading Frames (ORFs) were predicted using Prodigal [26] with default parameters but the predicted ORFs were excluded if they spanned a sequencing gap region. The predicted bacterial protein sequences were searched against the GenBank database [27] and the Clusters of Orthologous Groups (COG) databases using BLASTP. The tRNAScanSE tool [28] was used to find tRNA genes, whereas ribosomal RNAs were found by using RNAmmer [29] and BLASTn against the GenBank database. Signal peptides and numbers of transmembrane helices were predicted using SignalP [30] and TMHMM [31], respectively. ORFans were identified if their BLASTP *E*-value was lower than 1e-03 for alignment length greater than 80 amino acids. If alignment lengths were smaller than 80 amino acids, we used an *E*-value of 1e-05. Such parameter thresholds have already been used in previous works to define ORFans. Artemis [32] and DNA Plotter [33] were used for data management and visualization of genomic features, respectively. Mauve alignment tool (version 2.3.1) was used for multiple genomic sequence alignment [34].

To estimate the mean level of nucleotide sequence similarity at the genome level between *B. jeddahensis* sp nov. strain JCE^T^ and nine other members of the genus Bacillus, we use the Average Genomic Identity of orthologous gene Sequences (AGIOS) program. Briefly, this software combines the Proteinortho software [35] to detect orthologous proteins between genomes compared on a pair-wise basis, then retrieves the corresponding genes and determines the mean percentage of nucleotide sequence identity among orthologous ORFs using the Needleman-Wunsch global alignment algorithm. Moreover, we used Genome-to-Genome Distance Calculator (GGDC) web server available at (http://ggdc.dsmz.de) to estimate of the overall similarity among the compared genomes and to replace the wet-lab DNA-DNA hybridization (DDH) by a digital DDH (dDDH) [36, 37]. GGDC 2.0 BLAST+ was chosen as alignment method and the recommended formula 2 was taken into account to interpret the results.

### Genome properties

**Fig. 6:**
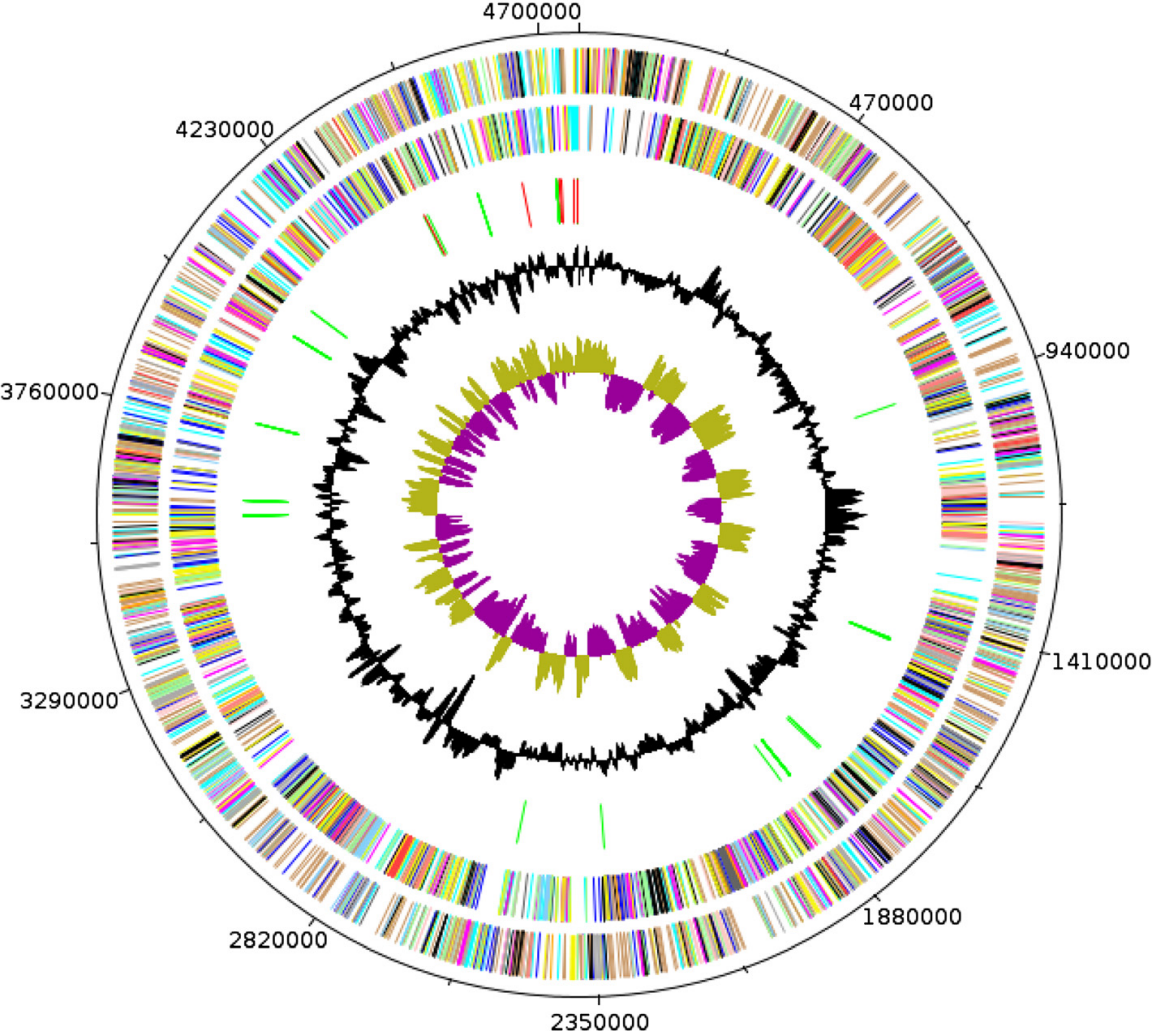
Graphical circular map of the chromosome. From outside to the center: Genes on the forward strand colored by COG categories (only genes assigned to COG), genes on the reverse strand colored by COG categories (only gene assigned to COG), RNA genes (tRNAs green, rRNAs red), G + C content and GC skew. Purple and olive indicating negative and positive values, respectively

The genome 4,762,944 bp long (1 chromosome, but no plasmid) with a 39.42 % G + C content (Fig. 6 and Table 3). It is composed of 149 contigs. Of the 4,741 predicted genes, 4,654 were protein-coding genes and 98 were RNAs including 6 rRNA (1 gene is 16S rRNA, 1 gene is 23S rRNA and 5 genes are 5S rRNA). A total of 3,410 genes (71.92 %) were assigned a putative function (by COGs or by NR blast) and 147 genes were identified as ORFans (3.17 %). The distribution of genes into COGs functional categories is presented in Table 4. The properties and statistics of the genome are summarized in Tables 3 and 4.

**Table 3:** Nucleotide content and gene count levels of the genome

Attribute	Genome (total)	
	Value	% of total^a^
Size (bp)	4,762,944	100
G + C content (bp)	1,876,599	39.42
Coding region (bp)	4,065,588	85.36
DNA scaffolds	ND	
Total genes	4,741	100
RNA genes	98	2.07
Protein-coding genes	4,654	97.72
Pseudo genes	ND	
Genes in internal clusters	ND	
Genes with function prediction	3,410	71.92
Genes assigned to COGs	2,902	61.21
Genes with Pfam domains	ND	
Genes with peptide signals	93	1.96
Genes with transmembrane helices	1,301	27.44
CRISPR repeats	ND	

^a^The total is based on either the size of the genome in base pairs or the total number of protein coding genes in the annotated genome. *ND* not determined

**Table 4:** Number of genes associated with the 25 general COG functional categories

Code	Value	% of total^a^	Description
J	179	3.86	Translation
A	0	0	RNA processing and modification
K	342	7.38	Transcription
L	195	4.21	Replication, recombination and repair
B	1	0.02	Chromatin structure and dynamics
D	39	0.84	Cell cycle control, mitosis and meiosis
Y	0	0	Nuclear structure
V	81	1.75	Defense mechanisms
T	255	5.5	Signal transduction mechanisms
M	192	4.14	Cell wall/membrane biogenesis
N	57	1.23	Cell motility
Z	0	0	Cytoskeleton
W	0	0	Extracellular structures
U	48	1.04	Intracellular trafficking and secretion
O	119	2.57	Posttranslational modification, protein turnover, chaperones
C	224	4.83	Energy production and conversion
G	300	6.48	Carbohydrate transport and metabolism
E	425	9.17	Amino acid transport and metabolism
F	89	1.92	Nucleotide transport and metabolism
H	131	2.83	Coenzyme transport and metabolism
I	139	3	Lipid transport and metabolism
P	261	5.63	Inorganic ion transport and metabolism
Q	82	1.77	Secondary metabolites biosynthesis, transport and catabolism
R	500	10.79	General function prediction only
S	348	7.51	Function unknown
-	1223	26.4	Not in COGs

^a^The total is based on the total number of protein coding genes in the annotated genome

### Insights from the genome sequence

**Fig. 7:**
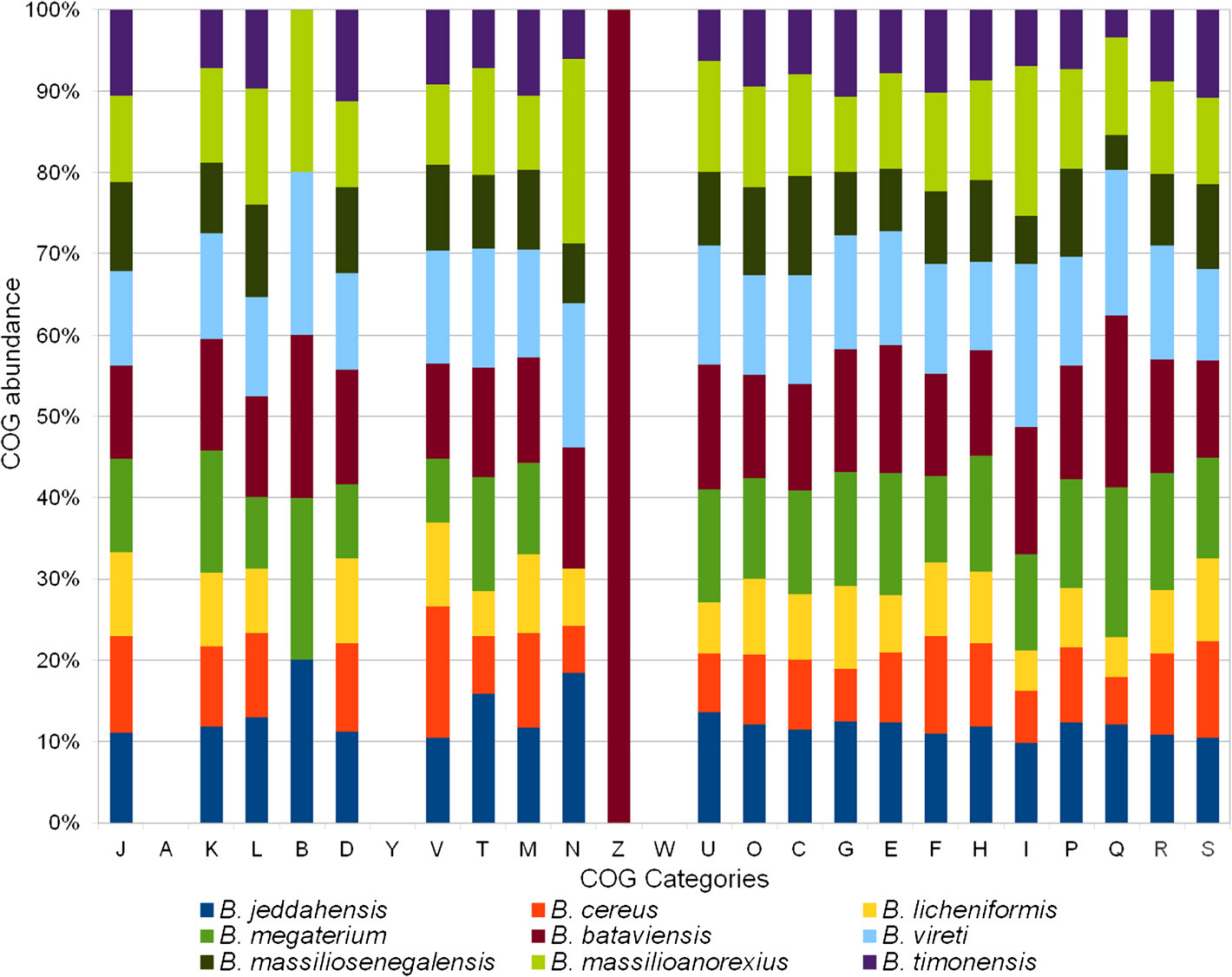
Distribution of predicted genes of *B. jeddahensis* and eight other *Bacillus* species into COG categories

Here, we compared the genome of *B. jeddahensis* strain JCE^T^ with those of “Bacillus massiliosenegalensis” strain JC6^T^*, “*Bacillus massilioanorexius*”* strain AP8^T^*, “*Bacillus timonensis” strain MM10403188*,*Bacillus cereus strain ATCC 14579*,*Bacillus megaterium strain DSM 319*,*Bacillus licheniformis strain ATCC 14580*,*Bacillus bataviensis strain DSM 15601^T^, Bacillus vireti DSM 15602^T^, Bacillus niacini JAM F8 and Bacillus niacini DSM 2923^T^ (Tables 5 and 6). The draft genome sequence of *B. jeddahensis* strain is larger in size than those of “B. massilioanorexius”, “B. timonensis”*,*B. licheniformis and B. niacini DSM 2923^T^ (4.76 vs 4.59, 4.66, 4.22 and 2.2 Mb, respectively), but smaller than those of “B. massiliosenegalensis”, B. cereus, B. megaterium*,*B. bataviensis*,*B. vireti and B. niacini JAM F8 (4.76 vs 4.97, 5.43, 5.10, 5.37, 5.29 and 6.37 Mb, respectively) (Table 5). *B. jeddahensis* has a lower G + C content than those of B. licheniformis*,*B. bataviensis*and B.vireti* (39.42 vs 46.19, 39.6 and 39.74 %, respectively) and higher than those of “B. massiliosenegalensis”*,”*B. massilioanorexius”*,”*B. timonensis”*,*B. cereus*,*B. megaterium*,*B. niacini JAM F8 and B. niacini DSM 2923^T^ (39.42 vs 37.58, 34.10, 37.28, 35.29, 38.13, 37.83 and 38.30 %, respectively) (Table 5). As it was reported recently that the G + C content varies no more than 1 % within species [38] and because the strain JCE^T^ differs from most of the other closely strains by more than 1 % in G + C content, this might provide an additional argument for the new taxon described herein. The protein content of *B. jeddahensis* is higher than those of “B. massilioanorexius”*, “*B. timonensis”*,*B. licheniformis and B. niacini DSM 2923^T^ (4654 vs 4436, 4647, 4173 and 2184, respectively) but lower than those of “B. massiliosenegalensis”*,*B. cereus*,*B. megaterium*,*B. bataviensis*, B.vireti* andB. niacini JAM F8 (4654 vs 4935, 5231, 5100, 5207, 5092 and 6103, respectively) (Table 6). The distribution of genes into COG categories was not entirely similar in all the nine compared genomes (Fig. 7). In addition, *B. jeddahensis* shares 2075, 1786, 1930, 1729, 1894, 1715, 2494, 2433, 2404 and 914 orthologous genes with those of “B. massiliosenegalensis”*, “*B. massilioanorexius”*, “*B. timonensis”*,*B. cereus*,*B. megaterium*,*B. licheniformis*,*B. bataviensis*,*B. vireti*,*B. niacini JAM F8 and B. niacini DSM 2923^T^, respectively. Among compared genomes except *B. jeddahensis*, AGIOS values range from 64.44 between *B.cereus* and B. licheniformis to 83.91 % between B. niacini JAM F8 and B. niacini DSM 2923^T^. When *B. jeddahensis* was compared to other species, AGIOS values range from 65.50 with B. licheniformis to 78.49 % with B. bataviensis (Table 6). dDDH estimation of the strain JCE^T^ against the compared genomes ranged between 19.50 to 28.10. These values are very low and below the cutoff of 70 %, thus confirming again the new species status of the strain JCE^T^. Table 5 summarizes the number of orthologous genes and the average percentage of nucleotide sequence identity between the different genomes studied.

**Table 5:** Genomic comparison of *B. jeddahensis* sp. nov., strain JCE^T^ with other *Bacillus* species. Species and strain names, GenBank genome accession numbers, sizes and G + C contents

Species	Strain	Genome accession number	Genome size (Mb)	G + C content
*Bacillus jeddahensis*	JCE^T^	CCAS00000000	4.76	39.42
“*Bacillus massiliosenegalensis*”	JC6^T^	CAHJ00000000	4.97	37.58
“*Bacillus massilioanorexius*”	AP8^T^	CAPG00000000	4.59	34.10
“*Bacillus timonensis*”	MM10403188	CAET00000000	4.66	37.28
*Bacillus cereus*	ATCC 14579	NC_004722	5.43	35.29
*Bacillus megaterium*	DSM 319	NC_014103	5.10	38.13
*Bacillus licheniformis*	ATCC 14580	NC_006270	4.22	46.19
*Bacillus bataviensis*	DSM 15601^T^	AJLS00000000	5.37	39.60
*Bacillus vireti*	DSM 15602^T^	ALAN00000000	5.29	39.74
*Bacillus niacini*	JAM F8	BAWM00000000	6.37	37.83
*Bacillus niacini*	DSM 2923^T^	JRYQ00000000	2.20	38.30

Bold numbers indicate numbers of proteins per genome

**Table 6:** Genomic comparison of *B. jeddahensis* sp. nov., strain JCE^T^ with other *Bacillus* species. Numbers of orthologous proteins shared between genomes (upper right triangle), average percentage similarity of nucleotides corresponding to orthologous proteins shared between genomes (lower left triangle)

	JCE^T^	JC6^T^	AP8^T^	MM10403188	ATCC 14579	DSM 319	ATCC 14580	DSM 15601^T^	DSM 15602^T^	JAM F8	DSM 2923^T^
JCE^T^	**4654**	2075	1786	1930	1729	1894	1715	2494	2433	2404	914
JC6^T^	69.86	4935	1858	1960	1669	1862	1746	2118	2132	2169	836
AP8^T^	68.13	68.67	4436	1726	1674	1778	1636	1848	1854	1911	711
MM10403188	68.48	68.73	68.49	4647	1631	1811	1691	2092	2047	2103	829
ATCC 14579	66.99	67.35	67.83	67.99	5231	1837	1678	1776	1792	1837	726
DSM 319	67.26	67.43	67.80	68.24	67.94	5100	1871	1978	1941	2033	805
ATCC 14580	65.50	65.33	64.32	65.72	64.44	66.06	4173	1761	1794	1784	716
DSM 15601^T^	78.49	69.51	67.94	68.53	66.69	67.18	65.54	5207	2699	2639	989
DSM 15602^T^	77.41	69.37	67.69	68.32	66.58	66.97	65.61	79.72	5092	2528	956
JAM F8	74.84	70.04	68.49	68.90	67.21	67.42	65.18	74.95	74.98	6103	1102
DSM 2923^T^	74.17	69.58	68.48	68.64	67.05	67.39	65.54	74.55	74.46	83.91	2184

Bold numbers indicate numbers of proteins per genome

## Conclusions

On the basis of phenotypic characteristics (Additional file 1: Table S1), phylogenetic position (Fig. 1), genomic analyses (taxonogenomics) (Table 5) and GGDC results, we formally propose the creation of Bacillus*jeddahensis* sp. nov*.* that contains the strain JCE^T^. This strain has been found in obese human feces collected from Jeddah, Saudi Arabia.

## Description of Bacillus*jeddahensis* sp. nov. strain JCE^T^

Bacillus*jeddahensis* (jed.dah. en′sis L. gen. neutr. n. *jeddahensis*, pertaining to, or originating from Jeddah, the capital of Saudi Arabia, where the type strain was isolated).

*B. jeddahensis* is a Gram-positive. Optimal growth is achieved aerobically. But growth is also observed in microaerophilic or anaerobic conditions. Growth occurs on axenic media between 28 and 45 °C, with optimal growth observed at 37 °C. Cells stain Gram-positive, are rod-shaped, endospore-forming and non-motile with a mean diameter of 0.87 μm (range 0.75 to 0.95 μm) and a mean length of 4.1 μm (range 3.8 to 4.7 μm). Colonies are smooth grey and 0.4–0.5 mm in diameter on blood-enriched Columbia agar.

Catalase negative, oxidase positive. A positive reaction is obtained for D-arabinose, L-arabinose, D-xylose, D-glucose, D-fructose, D-mannose, N-acetylglucosamine, esculin, D-maltose, D-trehalose, and weak reaction for D-melezitose. Negative reactions are obtained for the remaining carbohydrate tests (i.e. glycerol, erythritol, D-ribose, L-xylose, D-adonitol, methyl-β-D-xylopyranoside, D-galactose, L-sorbose, L-rhamnose, dulcitol, inositol, D-mannitol, D-sorbitol, methyl-α-D-mannopyranoside, methyl-α-D-glucopyranoside, amygdalin, arbutin, salicin, D-cellobiose, D-lactose, D-melibiose, D-saccharose, inulin, D-raffinose, amidon, glycogen, xylitol, gentiobiose, D-turanose, D-lyxose, D-tagatose, D-fucose, L-fucose, D-arabitol, L-arabitol, potassium gluconate, potassium 2-ketogluconate and potassium 5-ketogluconate). Positive reactions are observed for esterase (C 4), esterase lipase (C 8), acid phosphatase, naphthol-AS-BI-phosphohydrolase and β-glucosidase. Negative reactions are obtained for alkaline phosphatase, lipase (C 14), leucine arylamidase, valine arylamidase, cystine arylamidase, trypsin, α-chymotrypsin, α-galactosidase, β-galactosidase, β-glucuronidase, α-glucosidase, N-acetyl-β-glucosaminidase, α-mannosidase and α-fucosidase. Nitrates are reduced to nitrites, the urease reaction, indole production, arginine dihydrolase and gelatin hydrolysis are negative, the following carbon sources are assimilated: D-glucose, D-mannose, N-acetylglucosamine and D-maltose, and the following carbon sources were not assimilated: L-arabinose, D-mannitol, potassium gluconate, capric acid, adipic acid, malic acid, trisodium citrate and phenylacetic acid. *B. jeddahensis* is susceptible to imipenem, doxycyclin amoxicillin, amoxicillin-clavulanate and gentamycin, but resistant to metronidazole, trimethoprim/sulfamethoxazole, rifampicin, vancomycin, erythromycin, ceftriaxone, ciprofloxacin and benzylpenicillin.

The G + C content of the genome is 39.42 %. The 16S rRNA and genome sequences are deposited in GenBank under accession numbers HG931339 and CCAS00000000, respectively. The type strain JCE^T^ (= CSUR P732 = DSM 28281) was isolated from the fecal flora of an obese man from Jeddah in Saudi Arabia.

## Additional file

Additional file 1: Table S1. Differential phenotypic characteristics between *B. jeddahensis* sp. nov. strain JCE^T^ and phylogenetically close *Bacillus* species.

## Abbreviations

URMITE: Unité de Recherchesur les Maladies Infectieuses et Tropicales Emergentes
CSUR: Collection de Souches de l’Unité des Rickettsies
DSM: Deutsche Sammlung von Mikroorganismen
MALDI-TOF MS: Matrix-assisted laser-desorption/ionization time-of-flight mass spectrometry
TE buffer: Tris-EDTA buffer
GGDC: Genome-to-Genome Distance Calculator
dDDH: digital DNA-DNA hybridization
