# Supplementary material for: Non contiguous-finished genome sequence and description of Bacillus jeddahensis sp. nov
Source: Stand Genomic Sci. 2015 Aug 1;10:47. doi: 10.1186/s40793-015-0024-y (PMC4572673; doi:10.1186/s40793-015-0024-y)
Supplement: Additional file 1: Table S1. — Differential phenotypic characteristics between B. jeddahensis sp. nov. strain JCET and phylogenetically close Bacillus species. [file 40793_2015_24_MOESM1_ESM.doc]

**Table S1.** Differential phenotypic characteristics between *B. jeddahense* sp. nov. strain JCE^T^ and phylogenetically close [*Bacillus*](http://dx.doi.org/10.1601/nm.4857) species.

| **Characteristic** | 1 | 2 | 3 | 4 | 5 | 6 | 7 | 8 |
| --- | --- | --- | --- | --- | --- | --- | --- | --- |
| Motility | - | + | + | + | + | + | - | + |
| **Production of** |  |  |  |  |  |  |  |  |
| Nitrate reductase | + | + | + | var | na | + | +/w | + |
| Urease | - | - | - | - | - | - | +/w | - |
| Indole | - | - | - | - | - | - | + | - |
| Arginine dihydrolase | - | - | - | - | na | - | na | - |
| Gelatin hydrolysis | - | + | + | - | + | - | + | var |
| **Utilization of** |  |  |  |  |  |  |  |  |
| Glycerol | - | w | - | - | - | - | + | var |
| Erythritol | - | - | - | - | - | - | na | - |
| D-arabinose | + | - | - | - | - | - | na | - |
| L-arabinose | + | - | - | - | - | - | + | - |
| D-ribose | - | w | w | +/w | + | - | var | var |
| D-xylose | + | - | - | - | - | - | + | + |
| L-xylose | - | - | - | - | - | - | na | - |
| D-adonitol | - | - | - | - | - | - | na | - |
| Methyl-β-D-xylopyranoside | - | - | - | - | - | - | na | na |
| D-galactose | - | + | w | - | - | - | + | +/w |
| D-glucose | + | + | + | + | + | + | + | + |
| D-fructose | + | + | + | + | + | - | + | + |
| D-mannose | + | + | + | + | + | - | na | + |
| L-sorbose | - | - | - | - | - | - | na | - |
| L-rhamnose | - | - | - | - | w | - | na | - |
| Dulcitol | - | - | - | - | - | - | na | - |
| Inositol | - | - | - | - | - | - | na | na |
| D-mannitol | - | + | + | - | + | - | +/w | +/w |
| D-sorbitol | - | - | - | - | - | - | na | + |
| Methyl-α-D-mannopyranoside | - | +/w | w | - | - | - | na | - |
| Methyl-α-D-glucopyranoside | - | +/w | w | - | - | - | na | na |
| N-acetylglucosamine | + | + | + | + | na | + | na | + |
| Amygdalin | - | - | - | - | - | - | + | +/w |
| Arbutin | - | +/w | - | - | - | - | na | var |
| Aesculin | + | + | + | + | w | + | na | + |
| Salicin | - | w | - | + | w | - | var | - |
| D-cellobiose | - | + | - | - | w | + | var | +/w |
| D-maltose | + | + | + | + | - | + | + | + |
| D-lactose | - | + | - | + | - | - | na | - |
| D-melibiose | - | +/w | - | + | - | - | + | - |
| D-saccharose | - | +/w | + | + | + | - | na | na |
| D-trehalose | + | + | + | - | + | + | na | + |
| Inulin | - | +/w | - | + | - | - | na | - |
| D-melezitose | +/w | + | - | + | - | - | var | - |
| D-raffinose | - | + | - | +/w | - | - | - | - |
| Amidon (Starch) | - | - | + | +/w | na | - | - | - |
| Glycogen | - | - | + | + | - | - | - | - |
| Xylitol | - | - | - | - | - | - | na | - |
| Gentiobiose | - | + | - | - | w | - | var | +/w |
| D-turanose | - | + | - | +/w | - | - | + | - |
| D-lyxose | - | - | - | - | - | - | na | var |
| D-tagatose | - | - | - | - | - | - | na | - |
| D-fucose | - | - | - | - | - | - | na | - |
| L-fucose | - | +/w | w | - | - | - | - | - |
| D-arabitol | - | - | - | - | - | - | na | - |
| L-arabitol | - | - | - | - | - | - | na | - |
| Potassium gluconate | - | - | w | - | - | - | na | na |
| Potassium 2-ketogluconate | - | - | - | - | - | - | na | - |
| Potassium 5-ketogluconate | - | - | - | - | - | - | na | var |
| **Habitat** | Human gut | Soil | Soil | Soil | Human gut | Human gut | Soil | Soil |

Strain: 1, [*Bacillus*](http://dx.doi.org/10.1601/nm.4857) *jeddahense* JCE^T^; 2, [*Bacillus* *bataviensis*](http://dx.doi.org/10.1601/nm.4879) DSM 15601^T^; 3, [*Bacillus* *vireti*](http://dx.doi.org/10.1601/nm.5005) DSM 15602^T^; 4, [*Bacillus* *drentensis*](http://dx.doi.org/10.1601/nm.4898) DSM 15600 ^T^; 5, “[*Bacillus* *massilioanorexius*](http://dx.doi.org/10.1601/nm.24651)” AP8 ^T^; 6, “[*B. massiliosenegalensis*](http://dx.doi.org/10.1601/nm.24641)” JC6 ^T^; 7, [*B. niacini*](http://dx.doi.org/10.1601/nm.4951) DSM 2923^T^; 8, [*B. novalis*](http://dx.doi.org/10.1601/nm.4952) LMG 21837^T^

var: variable, +: positive result, -: negative result, na: data not available, w: weak positive result.
